# Event Detection System Based on User Behavior Changes in Online Social Networks: Case of the COVID-19 Pandemic

**DOI:** 10.1109/ACCESS.2020.3020391

**Published:** 2020-08-31

**Authors:** Renata Lopes Rosa, Marielle Jordane De Silva, Douglas Henrique Silva, Muhammad Shoaib Ayub, Dick Carrillo, Pedro H. J. Nardelli, Demóstenes Zegarra Rodríguez

**Affiliations:** 1 Department of Computer ScienceUniversidade Federal de Lavras (UFLA) Lavras 37200 Brazil; 2 Department of Electrical EngineeringChulalongkorn University 26683 Bangkok 10330 Thailand; 3 School of Energy SystemsLappeenranta–Lahti University University of Technology 53850 Lappeenranta Finland

**Keywords:** Event detection, online social networks, affective analysis, natural language processing, COVID-19

## Abstract

People use Online Social Networks (OSNs) to express their opinions and feelings about many topics. Depending on the nature of an event and its dissemination rate in OSNs, and considering specific regions, the users’ behavior can drastically change over a specific period of time. In this context, this work aims to propose an event detection system at the early stages of an event based on changes in the users’ behavior in an OSN. This system can detect an event of any subject, and thus, it can be used for different purposes. The proposed event detection system is composed of the following main modules: (1) determination of the user’s location, (2) message extraction from an OSN, (3) topic identification using natural language processing (NLP) based on the Deep Belief Network (DBN), (4) the user behavior change analyzer in the OSN, and (5) affective analysis for emotion identification based on a tree-convolutional neural network (tree-CNN). In the case of public health, the early event detection is very relevant for the population and the authorities in order to be able take corrective actions. Hence, the new coronavirus disease (COVID-19) is used as a case study in this work. For performance validation, the modules related to the topic identification and affective analysis were compared with other similar solutions or implemented with other machine learning algorithms. In the performance assessment, the proposed event detection system achieved an accuracy higher than 0.90, while other similar methods reached accuracy values less than 0.74. Additionally, our proposed system was able to detect an event almost three days earlier than the other methods. Furthermore, the information provided by the system permits to understand the predominant characteristics of an event, such as keywords and emotion type of messages.

## I. Introduction

The user behavior has been studied to examine the psychological antecedents of actions in various domains [1] for many years, and more recently to make recommendations [2], [3] and track diverse types of events [4]. The behavior of a person depends on many factors, such as his or her own health and well-being, and similarly, the behavior of the people can also depend on the public health status [5], [6].

Nowadays, online social networks (OSNs) are being used as a way of expressing feelings, emotions, and behavior [5], [6]. Thus, the OSNs provide an unprecedented amount of data, reflecting the behavior of the users [7]. However, analyzing the behavior of the users in an OSN is a complex task [8], and thus, some models to detect anomalies in the user behavior have been studied [9]. Some studies have focused particularly on the domain of user behavior analysis on social media for instance in the contexts of political events [10], [11], a diverse range of recommendation systems [12]–[14], public health [2], [15], communication network recommendations [16], and prediction of urban traffic trends [12], [17], among others.

Regarding the public health tracking status, some studies have focused on extracting messages in the OSNs for finding illness-related topics [2], [15]. Furthermore, OSNs have been used as an efficient resource to discover some disease outbreaks, in which it is possible to identify trends about a specific illness, correlating that OSN information to real-world illness patient data [18].

Currently, one of the most popular OSNs is Twitter, in which users share short messages. The data extracted from Twitter have been used in many studies [2], [15], [19] to identify possible trends. In addition, other similar OSNs are also used in different countries, such as Sina Weibo, the most popular micro-blog platform in China. In Weibo, it is also possible to classify disease-related information [20], [21]. To this end, the natural language processing (NLP) technique plays an important role. NLP is used for extracting situational information, such as advice, notifications, emotional support, doubt casting and criticizing, and counter-rumor [20]. In addition, different machine learning algorithms are used for illness type classification, such as Support Vector Machine (SVM), Naive Bayes (NB), and Random Forest (RF) [20], [22]. However, these algorithms do not reach an accuracy higher than 0.70 when they are applied in epidemic early detection solutions.

Different detection systems for infectious diseases caused by human influenza viruses have been proposed [19], [20], [23], [24], highlighting the importance of the early epidemic detection to minimize a negative impact [19]. The FluNearYou [25] is a web application that uses surveys to collect health statuses of individuals, associated with the data obtained from Google Flu Trend (GFT). Influenzanet [26] is a web application that collects real-time data about flu epidemics in European countries. Columbia Prediction of Infectious Diseases [27] is a web application that shows forecasts of seasonal flu, and HealthMap [28] is another infectious disease monitoring system. In [29], the focus of analysis is on some detection systems that use information about events impacting health, specifically, Dengue na Web [30], GripeNet [31], and Influweb [32] systems were analyzed. However, these disease detection systems are very specific, and do not cover other types of similar diseases. In addition, most of these studies are limited to investigating the content of questions and responses about a specific virus, not addressing the full potential of the data obtained from the OSN. Besides, they treat only local or regional events, not considering the potential spatial correlation between different geographical locations, such as the links of events in big cities of the world.

The users’ geographical location is also an important parameter to be collected from OSNs for tracing regional or global trends. However, geotags are affixed to only 1.5%–3.2% of user locations in OSNs [33]. Recently, studies have used the Social Triangulation (ST) [34] technique to identify locations of the OSN users who access certain community information [35].

The sentiment and affective analysis is another example of techniques that have been found useful to detect some medical conditions [2], [36] like depression or stress. Other medical conditions and diseases are also detected by extracting negative comments of the OSN, being associated with sadness or anger [2]. The affective or sentiment analysis can also make use of NLP, which helps to automatically extract meaning from texts, identifying themes or topics [37].

In general, the user behavior is influenced by personal experiences, and then, by events disseminated in OSNs [38]; therefore, the user behavior is a key parameter to detect events of different nature. However, the current studies do not analyze the relation between the user behavior change and possible future events. Although some works [24], [25], [30]–[32] detect peak events of diverse topics, there is a lack of studies related to the detection of a general event at an early stage by using the user behavior in OSNs.

In this context, our study proposes a method to extract data from Twitter and Weibo OSNs to analyze what features in the user behavior could be correlated to an important event. An infectious disease outbreak with global reach, the COVID-19, is used as a case study. Thus, this work highlights the relevance of using behavior change information to detect a new event related to the COVID-19 pandemic.

**FIGURE 1.**
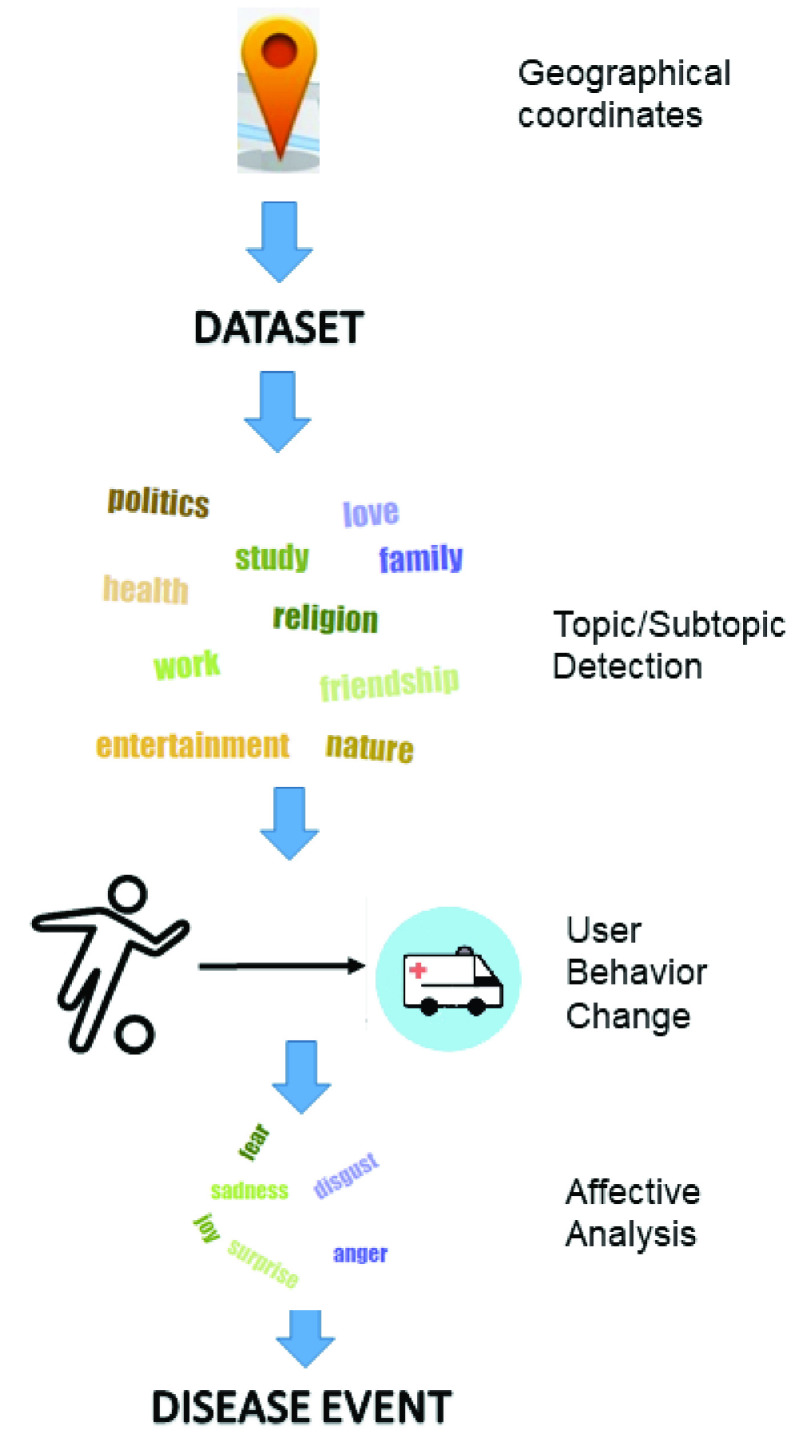
Outline of the proposed event detection system at an early stage based on changes in the user behavior.

Fig. 1 introduces the proposed event detection system in which the user location is first determined, and then, a dataset is built, and the topic and subtopic identification of the message are classified using the NLP technique. Later, the change of the topic of the user posts is flagged and the user behavior change is detected and analyzed. Depending on the change of topic, the event is discovered. Finally, an affective analysis is performed in the user message to identify the emotions and consequently, whether the event is positive or negative.

The main contributions of this paper are summarized as follows:

1) A method to implement an early event detection system based on the user behavior information that reaches an accuracy superior to related works.
2) A demonstration that user behavior changes in OSNs provide useful information to predict different event types; in our case study, the events are related to the COVID-19 pandemic at its early stages.
3) The performance validation of a deep belief network (DBN) and softmax regression in the NLP context.
4) The performance validation of a tree-convolutional neural network (tree-CNN) model for the affective analysis performed in this work.

The results obtained in the present case study show that the proposed event detection system at the early stages of the event showed a better performance than other related works [39], [40] and also that the event was determined a few days earlier than by similar solutions. In total, eight big cities around the world were used to analyze the user behavior in OSNs, capturing data from November, 2019 to March, 2020. Although this study focuses on an event related to a disease, it can be used for other topics, such as events related to crime, war, or politics, among others.

The rest of the paper is organized as follows. A more detailed literature review is presented in Section II. Section III introduces the methodology where the techniques used in the different modules of the proposed event detection system are described. The results of the proposed system and a comparative performance analysis are presented in Section IV. The conclusions of this work are presented in Section V.

## II. Literature Review

This section introduces and discusses the main studies in the literature about user behavior in OSNs, disease detection using data from OSNs and affective analysis, and topic detection based on NLP.

### A. User Behavior Analysis in OSN

In recent years, the user behavior has been studied either to understand the users’ physiologic behavior or the characterization of user activities and their usage patterns in OSNs [41]. To this end, the frequency at which people connect to social networks and the duration of staying connected have been measured [42], as well as the types and number of activities that the users perform on these websites. Thus, the user behavior analysis in OSNs can be applied for different purposes. Some studies [41], [42] have focused on improving the performance of content distribution systems by using user behavior information. In [43], authors used the user behavior information to discover anomalous activities and validate the reliability of the user profiles. Other studies [44], [45] have predicted the user behavior for discovering online social communities.

In the Twitter OSN, the user behavior can also be characterized in relation to the following activities: tweeting, retweeting, and commenting [46]. Other OSNs, such as Sina Weibo, have also been used [47], [48] to extract data and analyze the user behaviors, and then determine the impact of the user popularity on OSN websites. It is important to note that these studies do not explore the changes of topics posted by users.

It is also known that certain events can attract more public attention, which is demonstrated by the number of messages or communication interactions between people interested in such topics [49]. Thus, through the number of messages in OSNs, it is possible to measure the number of members related to potential events, and concerning specific regions. This helps to solve the problem of early event identification. Hence, the messages posted in an OSN represent valuable information to understand and predict the users’ behavior in a specific period of time and geographical location.

Different from the above-mentioned works, our study analyzes the relation between the user behavior in an OSN and possible events, identifying the occurrence of topic and subtopic changes, and the increase in the number of messages extracted from the OSN over a period of time.

### B. Disease Detection Using Data from an OSN

Currently, there are diverse solutions to detect different types of events using data from an OSN. However, because the focus of our case study is on disease detection, only works related to this subject are presented.

In [50], [51], the authors stated that the virality of a social media content, in the public health context, can depend on the users’ emotions and the disease type. Additionally, the number of followers can affect the propagation scale of the posted messages in OSNs [52], [53]. Thus, the greater the virality of a content, the easier its detection.

The virality of a post also depends on the geographical location of the users. An user from a big city can be more influential than users in smaller cities [54]. In the case of accidents or disasters, people usually share information more quickly and mostly with people close to the event [55].

Currently, there are many studies about flu-related disease detection [24] that are based on OSNs, in which the studies commonly classify tweets about actual flu cases and tweets that seem related to the flu but are not actually about the flu cases [56]. The posts in OSNs are also classified into health-related or unrelated ones, and they are further divided into local and national posts [57]. The accuracy of the model proposed in [56] to classify health-related or unrelated contents presents a high correlation reaching a Pearson correlation coefficient value of 0.9897. However, both these studies [56], [57] are limited to only two categories of classification.

Different frameworks for flu-related detection were proposed in [58], [59] based on machine learning algorithms. They extracted the actual influenza tweets and excluded the unrelated ones. In [58], a dataset built of posted tweets was filtered using the “influenza” keyword to obtain a set of only flu-related tweets. In the training phase, a specialist manually labeled each tweet as either positive or negative. A tweet was positive if the flu tweet was about the person who posted the tweet or about another person next to her/him and if the tweet was an affirmative sentence. In [58], the authors proposed a flu detection method using different machine learning algorithms, and this method showed a low performance in the classification of messages regarding the swine flu in 2009. An SVM-based classifier was also used in [59] for detecting a flu-like illness in Portugal by using Twitter. In the training and testing phase, a dataset was manually annotated with 650 features. The classifier used the Bag-of-Words feature representation. The results were compared with the reports of Influenzanet, which is a system that monitors Influenza in Europe. The correlation coefficient between the results of the method proposed in [59] and Influenzanet reached 0.89.

Cui *et al.* [60] presented a similar method to predict flu trends from a Chinese OSN, in which 50,000 posts were selected for manual annotation, with labels ’sick’ and ’not sick’. The authors concluded that the SVM algorithm reached the best performance. The method predicted the flu trend five days earlier than the China Nation Influenza Center (CNIC). However, similarly to other studies [56], [57], the method was limited to two classes.

In [61], machine learning techniques were applied to Arabic tweets by conducting a sentiment analysis and trying to improve the accuracy of the classification of the disease. In this work, influenza-related tweets were collected, labeled, filtered, and analyzed by machine learning algorithms, such as Naive Bayes, SVM, Decision Trees, and K-Nearest Neighbor. The Naive Bayes classifier presented the best results, reaching an accuracy of 89.06%. In [62], a method to monitor the spread of influenza was presented in selected cities in real time; however, the study was limited to search influenza-related keywords in OSN messages. Other machine-learning-based methods were proposed in [20], [23], in which flu-related activities were predicted. The method proposed in [23] used data from different sources to obtain a better result. Data from Google searches, GFT, Twitter posts, and hospital visits were included. The method used machine learning algorithms, such as SVM, stacked linear regression, and AdaBoost with decision tree regression. The method was able to predict an event one week faster than the GFT site. Similarly, machine learning algorithms were used in [20] for classifying COVID-19-related information. The average accuracy of the classifiers was around 0.65.

Event detection solutions were proposed in [39], [40]. In [39], authors introduced a Multiscale Event Detection (MED) method based on Wavelets and using social media, which takes into account different temporal and spatial scales of events in the data. In addition, the authors proposed a second method called Local Event Detection via Locality Constraints (LED). The MED presents a better performance than the LED in detecting events of different scales, considering F-measure results. An event detector was proposed in [40], which dynamically updates a set of events. The MED [39] and the event detector described in [40] were chosen to be used in the performance assessment of the detection system proposed in the present study.

### C. Topic Detection and Affective Analysis

A method of topic detection based on NLP was used for COVID-19 prediction in [63] by applying a hybrid artificial intelligence (AI) model. The change in the infectious capacity of the virus was analyzed within a few days after the infection, and an improved susceptible-infected (ISI) model was proposed. The NLP module and the LSTM network were embedded in the ISI model to build a hybrid AI model for COVID-19 prediction. With the NLP and LSTM built into the hybrid AI model, the mean absolute percentage errors of the prediction results, considering the next six days, were 0.52%, 0.38%, 0.05%, 0.86% in Wuhan, Beijing, Shanghai, and nationwide, respectively. In [64], deep learning algorithms were used for NLP using a Contrastive Divergence (CD) algorithm, such as the Deep Belief Network (DBN) [65], which is composed of restricted Boltzmann machines (RBMs) [64].

Other two methods to detect topics were presented in [66]. The Soft Frequent Pattern Mining (SFPM) algorithm [67] and the BNGram method [68] were analyzed for topic detection. The SFPM [67] works on a frequent pattern mining approach and association rules [69] considering the simultaneous co-occurrences between any number of terms. The BNgram method [68] is based on finding emerging topics by considering the co-occurrences of n-grams instead of unigrams and analyzing the frequencies of terms in the current time slot and the preceding ones. Both the methods are used in the present work for comparison with the proposed topic detection method based on NLP.

Sentences can describe emotions and sentiments of the users, and they refer to intrinsic attractiveness or aversiveness of a subject, which can be an object, event, or situation [70]–[72]. Hence, the studies use the sentiment analysis to discover the opinions of the population about some specific situations. The opinions of Twitter users about flu vaccines were measured in [73]. A sentiment and affective analysis was performed in [74] to discover the user behavior in the nationwide lockdown caused by the COVID 19 outbreak in India. Another study used the sentiment analysis to predict the adoption of social distancing in the COVID-19 pandemic [75], in which 82 subjects participated by answering open-ended questions. The results suggested that when friends and peers behave responsibly, a person also adopts the same behavior. A risk prediction method was proposed in [76] to discover the number of infections and death cases related to COVID-19 and their impact on the economy. Another work about COVID-19–related discussions was presented in [77], in which a method based on a Long Short-Term Memory (LSTM) recurrent neural network was used for the sentiment classification of COVID-19 comments posted on OSNs.

The emotional valence of tweets was measured in [70]. The scores were normalized and the polarity of tweets was detected as being positive, negative, or neutral. Additionally, an affective analysis was performed using recurrent neural networks to label the emotion as anger, disgust, fear, joy, sadness, or surprise. The topic modeling was performed using an unsupervised machine learning method to identify topics over time. The most frequent words extracted from the tweets were outbreak, spread, health, confirm, death, city, report, first, world, travel, hospital, infect, SARS, mask, patient, and country. Another work introduced EmoMix [78], which is an emotion lexicon for compound emotion analysis. Both studies [70], [78] used the same emotions in the text message context. For this reason, the solutions presented in both studies [70], [78], are used for performance comparison purposes related to the system proposed in this study.

In this paper, affective analysis and topic detection are studied to obtain a model in order to increase the accuracy in relation to the existing works. It is important to note that the models are used in the text processing context.

## III. Methodology

This section presents the methodology of the proposed event detection system at the early stages of an event. The event detection system is based on changes in the users’ behavior, and it uses messages extracted from Twitter and Sina Weibo [79]. The user behavior was studied from November 15th, 2019 to March 15th, 2020.

Firstly, for the purposes of this case study, it is important to know the official data about the pandemic in the world. This information is relevant for the final performance assessment of the proposed system in the case study. For example, in Brazil, the first case of COVID-19 was confirmed on February 26, 2020. However, the first disease case in the world was identified in Wuhan, Hubei Province, People’s Republic of China, on December 1, 2019. Table 1 presents the dates of the first confirmed cases and deaths, according to the [80] caused by the COVID-19 in some countries studied in this work, such as China, the United States, Italy, Spain, the United Kingdom, Brazil, and Peru, considering the order of the arrival date of COVID-19 in each country. It is important to note that the data extracted from [80] are collected from the official bodies of each country.

**TABLE 1:** Dates of the First Confirmed Cases and Deaths Caused by COVID-19 in China, United States, Italy, Spain, United Kingdom, Brazil and Peru

Country	Date of first case	Date of first death
China	December 1, 2019	January, 11, 2020
United States	January 20, 2020	February 6, 2020
Italy	January 31, 2020	February 22, 2020
Spain	January 31, 2020	February 13, 2020
United Kingdom	January 31, 2020	March 05, 2020
Brazil	February 26, 2020	March 17, 2020
Peru	March 06, 2020	March 20, 2020

**FIGURE 2.**
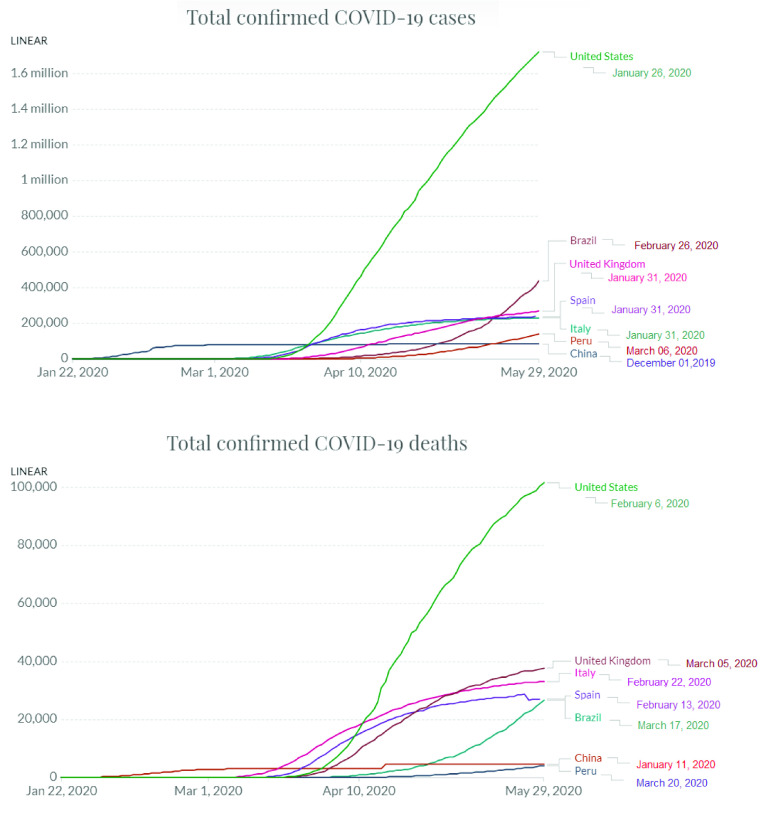
Number of confirmed cases and deaths related to COVID-19 in some countries from January to May, 2020.

In Fig. 2, the evolution of the numbers of both confirmed cases and deaths related to COVID-19 are shown. The data correspond to the period from January to May, 2020 [81]. It is important to note that in this study, posted messages were collected from the OSNs before the new coronavirus appeared in humans in the world. Thus, changes in the user behavior can be analyzed by studying user messages in OSNs.

**FIGURE 3.**
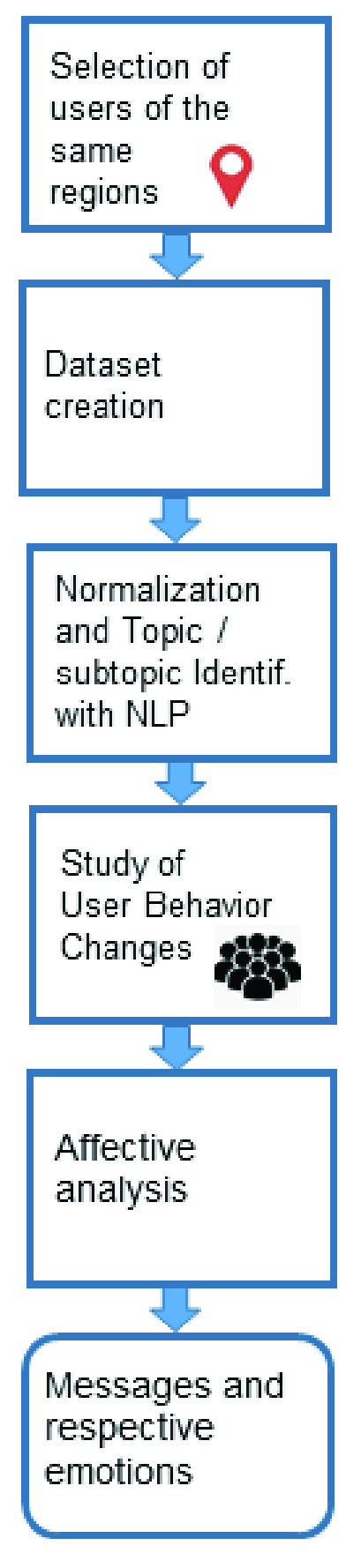
Methodology of the proposed Event Detection System based on Changes in the User Behavior in OSNs.

The methodology used to obtain the proposed event detection system is presented in Fig. 3. The first step of the proposed system is perform the selection of users of the same regions from Twitter and Weibo. If the user’s location is unknown, it is found by applying the ST technique. Thus, the system receives a continuous stream of messages as the input. A dataset is built using messages from OSN. The messages are normalized for discarding unnecessary messages, and the NLP technique is applied to determine the topics and subtopics of the messages. The DBN and softmax regression applied to NLP are responsible for automatically finding the subtopics. The study of user behavior changes in the OSNs is detected by a script. Thus, a new topic or subtopic reflects a change in the user behavior. At the end of the process, affective analysis is applied to the messages using the Tree-CNN algorithm to identify the positive and negative emotions related to possible events.

### A. Selection of Users of the Same Regions

As previously stated, the user messages within a same region are extracted. However, many users do not configure their location. In that case, the ST technique is used. Eight big cities around the world were selected for the study: Sao Paulo, Lima, and New York City in the Americas; Hong Kong and Shanghai in Asia; and Madrid, Milan, and London in Europe. In these cities, Twitter or Weibo is used as an OSN.

The users were chosen randomly in each region to capture data from a representative sampling in large-scale OSNs [82], in which at least a thousand users per region were selected. A total of 8023 users of OSN were analyzed in this work.

The methodology for selecting users of the same region involves extracting the “geo-tagged” tweets and Weibo data. As stated above, in the case of missing location, the ST technique is used, which involves four processes in relation to community assets, according to [34]: categorizing, cataloguing, collecting user information, and analyzing geographic characteristics and information patterns.

The categories of public and private organizations to set the community assets were identified and listed in Table 2. The categories mentioned in Table 2 were taken independently. According to related works [34], these community assets are commonly followed by OSN users; thus, these communities help to find the user location.

**TABLE 2:** Categories of Community Assets

Category	Description
Citizens’ Associations	Nonprofit organizations
Civic Services	Civic government and public services
Emergency Services	Response management services
Schools	Municipal school districts
Bars	Establishments of good cheer
Entertainment	Recreational businesses
Restaurants	Local food-serving establishments
Media	Local media, such as newspapers, newsletters, websites, radio, television

The Twitter and Weibo APIs were used for collecting the data, and this information was imported into tables. After this, the information was geocoded using tools of the Google Map search. The messages were collected and stored in a database, being separated by the regions.

ST technique was used in this work by the community assets, in which the online communities followed by users are extracted from OSN, and their locations are found by Google Maps using the API Picker library. The geo-location of the communities determine the location of the user. In general, the experiments show that 92% of the communities are related to the user location [34]. The communities are commonly indexed into Google Maps by their names. Thus, the geocoding is given by Google Maps using an API called Picker.

**FIGURE 4.**
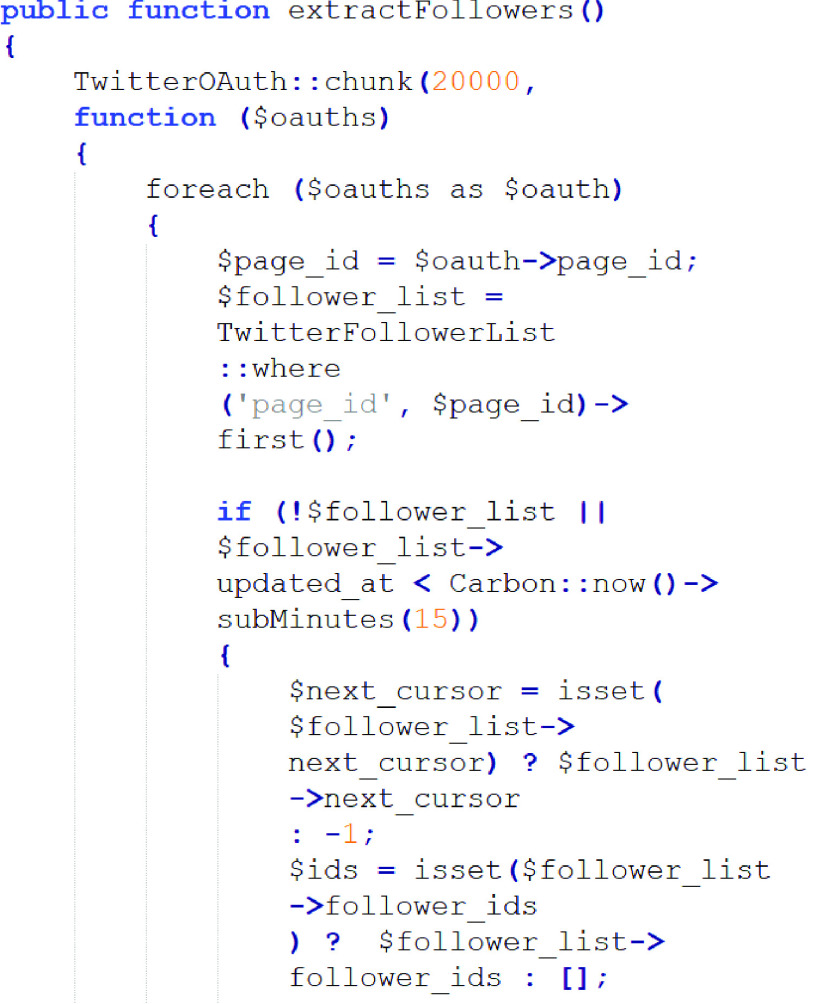
Code used to extract the communities followed by an user.

Fig. 4 shows the code for extracting the communities followed by an user in Twitter using the JSON-format data.

The Picker API is responsible for finding the latitude/longitude coordinates for determined geocoding, and in this study, the messages are organized by regions. Thus, geocoding is the parameter that helps to find the messages of a determined region. Related studies use the API Picker for the same purpose [83].

**FIGURE 5.**
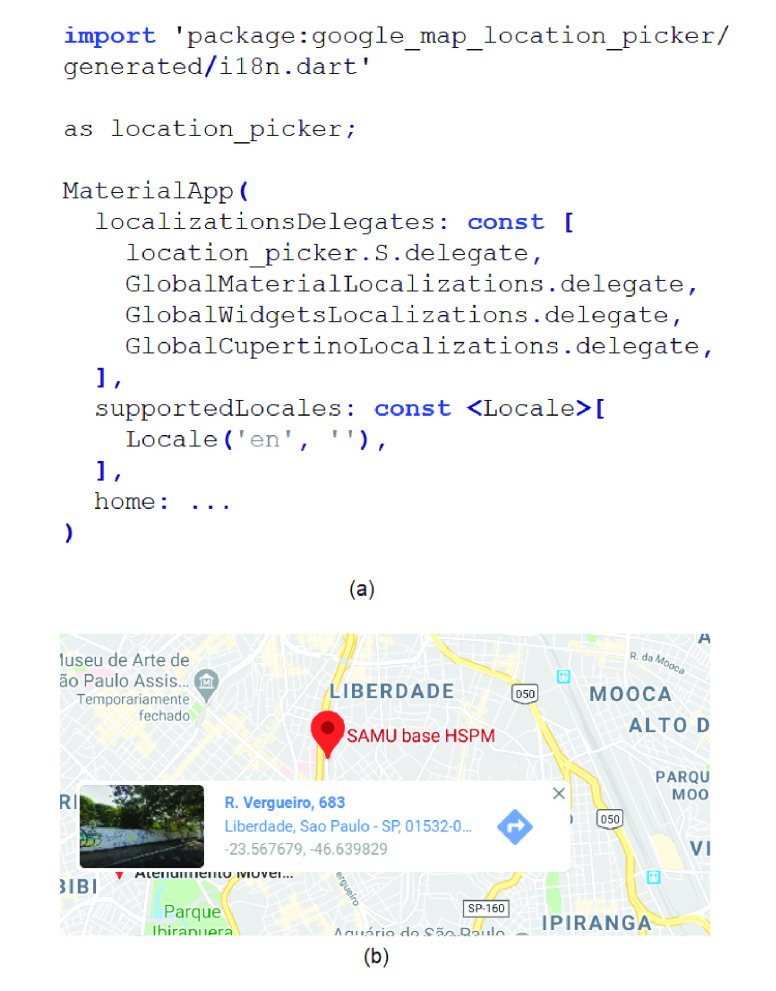
Community location found by: (a) API Picker and (b) Google Maps.

Fig. 5 shows an example of the geocoding of an emergency service followed by a person, in the region of Sao Paulo. Fig. 5 (a) presents the API Picker to extract the geo-location data, which be recorded in the database. Fig. 5 (b) shows the latitude and longitude data that were found by the Google Maps through the address of the emergency service used as example.

A method to examine an attribute-based model of the structuration of the local network was performed, creating a table of homophily scores with a standard $E$-$I$ index [84], in which the key parameter is the number of organizations that each user follows. Thus, the distribution of the users is analyzed, indicating different levels of embeddedness in the local information, also showing how many organizations each user follows, with the types of local information followers and information recipients.

In this work, five types of users are considered. These groups are defined according to the number of online local organizations that the users follow, which are the following. “Unique” means that the user follows an organization independent of the category introduced in Table 2, in this case, the organization is considered the unique recipient of the local information; “Low” means that the users follow only two organizations; “Moderate” means that the users follow from three to nine organizations; “High” means that the users follow from 10 to 49 organizations, and finally, “Extreme” means that the users follow more than 50 organizations in OSN.

In order to evaluate the performance of ST, the location information of the users that had the location configured in the OSN was collected and compared with the results given by the ST technique.

### B. Dataset Creation

In total, considering the eight analyzed cities, 18,597,314 messages were extracted from both OSNs used in this work. It is important to note that this study covers the Portuguese, Spanish, Italian, English, Cantonese, and Mandarin Chinese languages. With the data extracted, a dataset was built. Thus, each message was labeled with its respective topic and emotion by assessors. To this end, six assessors responsible for each language were assigned for this task, and they worked together with, on average, 64 volunteers. Thus, the assessors comprised 184 men and 208 women with ages ranging from 18 to 57 years old. This task was performed in approximately nine weeks, and during the same period some messages were simultaneously extracted.

It is noteworthy that the subtopics were classified automatically, and classification tasks by the assessors were not required.

### C. Text Preprocessing and Topic/Subtopic Identification by NLP

**FIGURE 6.**
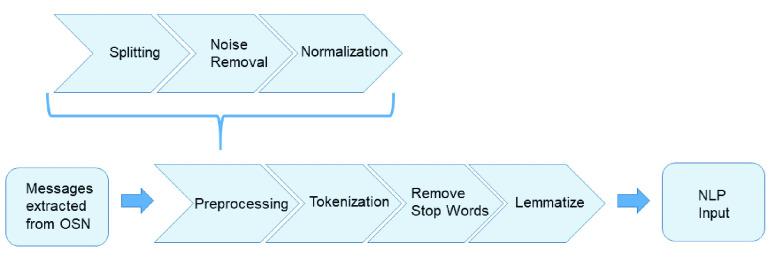
Block diagram of the steps involved to generate the input to NLP process.

Before the text is processed by the NLP, the messages must be treated and filtered as shown in Fig. 6.

As can be observed from Fig. 6, once the messages have been extracted from the OSNs, they go to the preprocessing step, which is divided into splitting, noise removal, and normalization. In these phases, the sentences are split and sent to the noise removal eliminating spam, bots, and ads using a Python program. Then, the normalization and spelling correction processes are applied. In the normalization module, the words of the message are unified; for example, in the case that the words are synonyms, they are considered a single word. The next step in the normalization is performed to correct misspellings. In the tokenization step, the hashtags are decomposed. For instance, the expression #prayformyMother is transformed into “pray for my mother”, being the sentence divided into tokens. Moreover, with the aim to improve the accuracy, we removed frequently occurring words, mostly known as stopwords that have no significant impact on the NLP and the affective analysis process. The lemmatization is carried out by using the Natural Language Toolkit (NLTK) package called Wordnet database. Finally, the messages are analyzed by NLP to detect the topics and subtopics of the message.

**FIGURE 7.**
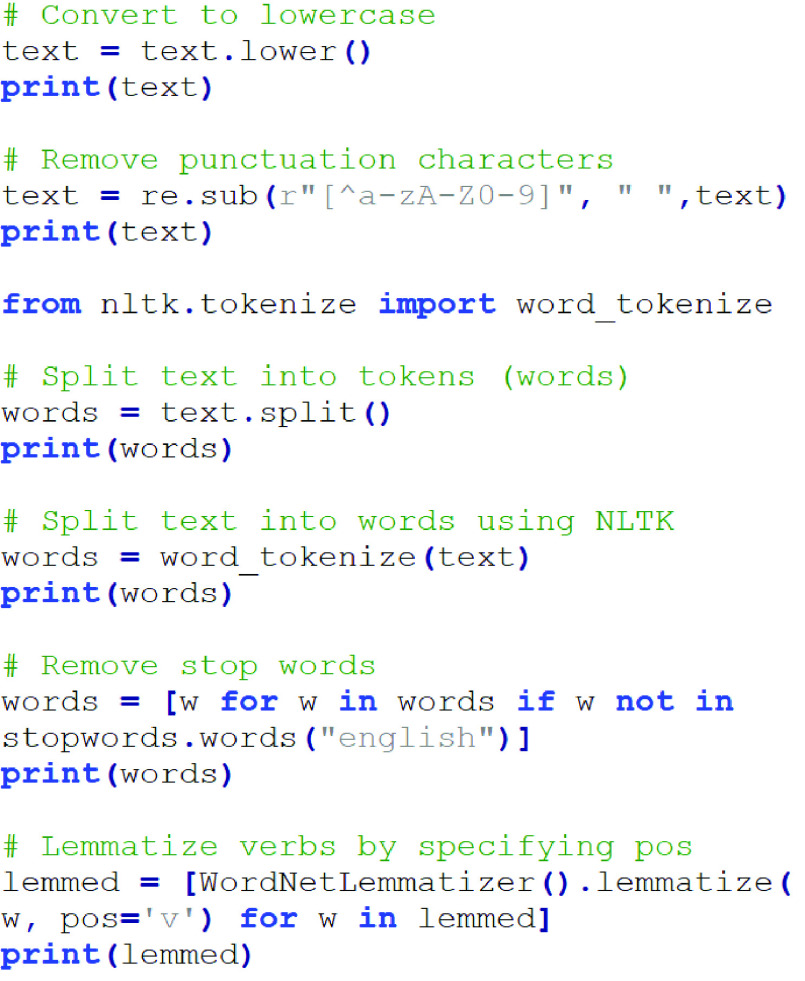
Steps from normalization to lemmatization using a Python script.

For a better illustration, Fig. 7 shows the steps from normalization up to lemmatization in a Python script.

It is important to note that in this work, different approaches of normalization, NLP, and affective analysis were performed, according to the language of the analyzed region. For this, specialists from different regions assisted in the translation phase.

The NLP algorithm was used for the topic and subtopic identification to help to identify the user behaviors. It is a rule-based algorithm and additionally, it uses the DBN and softmax regression for prediction of the subtopics.

The LiblineaR library [85] was used to classify the messages into different categories. The logistic regression classifier was used to calculate the probability outputs. In the logistic regression, the class $x$ has a separate vector $w_{x}$ of weights for all features. The higher the sum of the features of $t$, weighted by $w_{x}$, the greater the probability of the feature vector $t$ belonging to a class $x$. We have then

\begin{equation*} P(x|t;w_{x}) \alpha e^{\sum _{d}^{i=1}w_{xi}t_{i}}, \tag{1}\end{equation*}

where $t_{i}$ represents the $i$th feature and $w_{xi}$ represents the weight in the class $x$. The extracted information per message, in the form of features, is converted into a vectorial format with an Euclidean vector compatible with LiblineaR.

**FIGURE 8.**
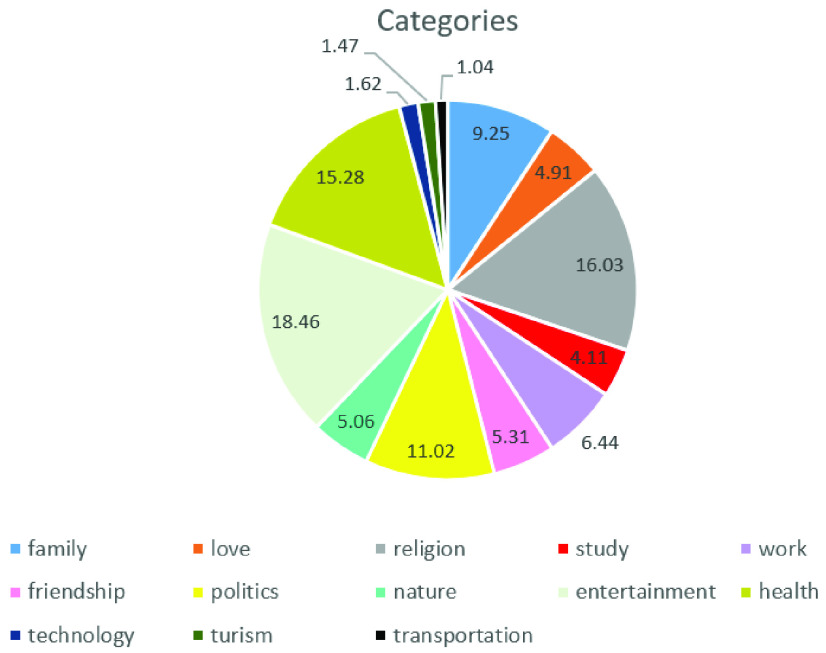
The most cited topics extracted from the sentences of OSN, in which the percentage of each category is represented.

The 10-fold cross validation method was used in both the training and testing phases. The topics to be identified were: family, love, religion, study, work, friendship, politics, nature, entertainment, and health. The topics were chosen according to the predominant themes found in the analyzed sentences from the OSNs, and limited to ten. Fig. 8 shows the percentage of the most cited topics extracted by the LiblineaR library, in which the percentage of each topic is represented. It is important to note that the topics with values lower than 2% were disregarded because they represent only a few sentences, and only ten topics were considered in the present work.

Later, there were 100 different subtopics that were discovered by the DBN and softmax regression, based on an unsupervised algorithm. Thus, it was not necessary to define the subtopics, but they were defined automatically, according to the modeling phase of the algorithm. The 100 most cited subtopics were considered in this specific case of coronavirus. However, the number of subtopics is not limited to 100, but it can vary depending on the content of the messages extracted from the OSN.

A ranking of the most cited subtopics for each topic is generated using the R package. This ranking is established according to the percentage of messages that each subtopic has considering the total messages of the topic. Some topics with their respective more cited subtopics are presented as follows.

• entertainment: sport (3.56), music (2.8).
• religion: pray (2.94).
• health: flu (3.48), fever (1.84), breathing (1,41).
• politics: manifestation (2.51).
• family: confraternization (1.46).

**FIGURE 9.**
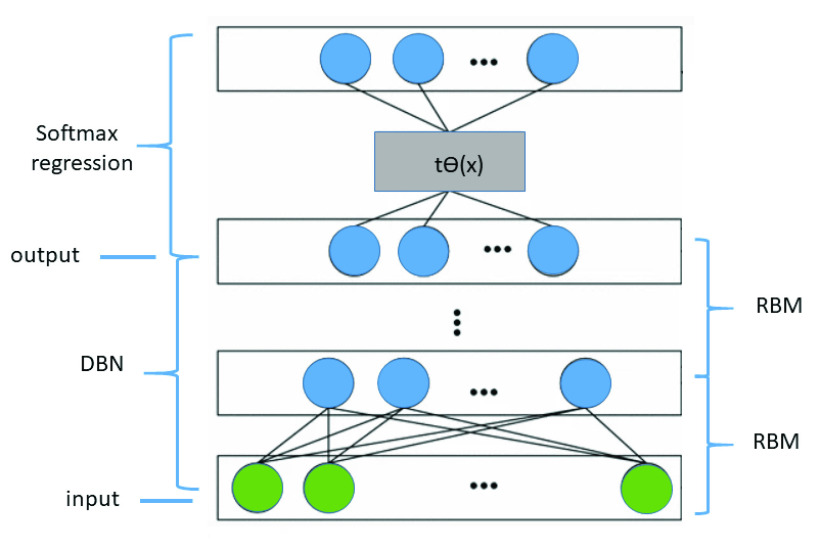
DBN algorithm topology with the softmax regression.

The task of text processing in the message can cause a sparse high-dimensional matrix computation problem. Thus, a DBN was introduced to resolve this problem. Firstly, the feature extraction was performed with the DBN, and after that, a softmax regression was employed to classify the message. In the DBN, the input layer is used to train the connection weights between the two layers, and the output layer offers the input of the next RBM. The softmax regression is used in the DBN, as can be seen in Fig. 9.

The input layer, which is the last one, is the message, and the output represents the feature learning results produced by the DBN. The generated output serves as the input of the softmax regression. The DBN is trained, and then, partial labeled data are used to train the softmax regression. Thus, the softmax regression receives weights, and for the softmax regression training, the input is $m$, the hypothesis function to compute the probability $p(y=t|m)$ for each message $m$ belonging to each subtopic $t$. For each classification $l$, it is assumed that the output is a one-dimensional vector of possible subtopics. To distinguish the parameters of the DBN, the function parameter is defined as $s\theta $. Thus, the output of the softmax regression ($h_{s\theta }$) is represented by:

\begin{align*} {\scriptscriptstyle h_{s\theta } = \left [{ \begin{array}{ll} p(y^{(i)} = 1)|m^{(i)};& s\theta \\ p(y^{(i)} = 2)|m^{(i)};& s\theta \\ \vdots &\quad \\ p(y^{(i)} = l)|m^{(i)};&s\theta \\ \end{array} }\right] = \frac {1}{{\sum \nolimits _{t = 1}^{l} {e^{{s\theta _{t}^{T} m^{(i)} }} } }}\left [{ \begin{array}{l} e^{{s\theta _{ 1}^{T} m^{(i)} }} \\ e^{{s\theta _{ 2}^{T} m^{(i)} }} \\ \vdots \quad \\ e^{{s\theta _{\text {l}}^{T} m^{(i)} }} \\ \end{array} }\right] }\tag{2}\end{align*}

Once the DBN and softmax regression training is finished, the labeled data for fine-tuning are used for all the parameters. In this work, the gradient descent algorithm and the limited-memory Broyden–Fletcher–Goldfarb–Shanno (L-BFGS) algorithm were used to optimize the cost function, which is represented by:

\begin{align*} { J(s\theta) \!\!=\!\! \frac {-1}{m}\sum \limits _{i = 1}^{m} {\sum \limits _{t = 1}^{k} {y_{t}^{i} \log (t_{s \theta t})} } \!\!+\!\! \frac {\lambda }{2}\sum \limits _{i = 1}^{l} {\sum \limits _{t = 0}^{n} {(s\theta {}_{it})^{2} } } \!+\! \frac {\lambda }{2}\sum \limits _{p = 1}^{q - 1} {\theta _{p}^{2} } }\!\! \\ \tag{3}\end{align*}

where $k$ is the number of subtopics, $m$ represents the number of previous computations to be stored, $y_{t}^{(i)}$ is the label of $t$ class, $t_{s \theta t}$ is the output of the softmax regression, $s_{ \theta it}$ represents the parameters of the model, $\lambda $ is the penalty factor, and $\theta _{p}$ is the weight of the RBMs in the DBN.

### D. Study of User Behavior Changes

**FIGURE 10.**
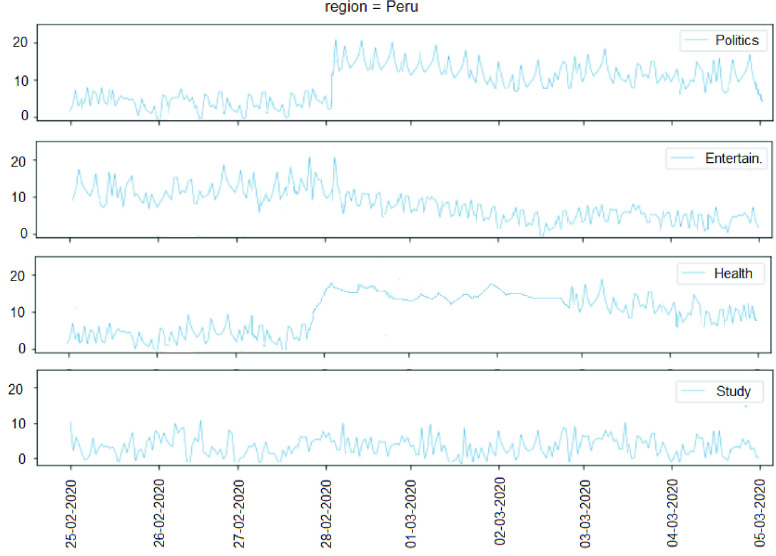
Monitoring the number of messages of four topics day by day in Lima city.

After the topic and subtopic classification by the NLP technique has been performed, the topics of the messages of an user are compared. Studies [86] show that people behave in a homogeneous behavior in an OSN; a person has tastes and she/he usually expresses herself/himself showing the tastes among the OSN friends. Thus, we studied the user behavior during each day to detect the period of time that the user behavior began to change. The messages of a day of a group of users from some region are compared with the messages of the last day, and the topics and subtopics are represented in a graph day by day. All the topics are analyzed to discover if there are common topics that have changed in the majority of the users’ messages from the same city. Fig. 10 shows the monitoring of four topics day by day in the region of Lima, Peru, using a script developed in Python. As can be observed in Fig. 10, a change in the users’ behavior occurred after February, 28th.

The script was used to automatically detect the changes in topics per region. In case the topics are different, the new topic is recorded and the subtopics of the messages are extracted. The subtopics that are equal are quantified. The largest number of equal subtopics represents a new potential event that is about to occur. After this process, an affective analysis of the messages is performed to know if the new event will be negative or positive.

### E. Affective Analysis

In this work, many machine learning algorithms were tested for performing the affective analysis, such as the SVM, RF, decision tree (DT), CNN, and Tree-CNN. The hierarchical CNN model, the Tree-CNN, presented the best performance results for the affective analysis, when compared with the other listed ones.

The Tree-CNN starts as a single root node, and after that, new hierarchies are generated to accommodate new classes. The steps performed by the Tree-CNN are initially training the network for classifying the data into $N$ classes, and when a new class appears, it is inserted into the networks. Thus, the network grows by adding a new leaf/branch node to the current structure.

A three-dimensional matrix is built from the output layer, in which there are the children of the root node, the number of new classes, and the number of sample texts per class. The softmax likelihood is used in the matrix.

The main word in the sentence is chosen as a root, and with the other words, one of the following actions occurs:

• Add the new word to an existing child node. The softmax outputs is represented by $o1$, $o2$, and $o3$. If $o1$ is greater than the next value $o2$ with a threshold $\alpha $, then the new word word indicates a strong association with a particular child node.
• Merge two child nodes in order to form a new child node and add the new word to this node. In the case that the new word does not have a likelihood value or all child nodes are full, then the network structure grows. In the case that there are more than one child node in which the new word has a strong likelihood, then it can combine them to form a new child node. This occurs when $o1 - o2 < \alpha $ and $o2 - o3 > \beta $.
• Add the new word as being a new child node. In the case the new word does not have a likelihood that is greater than others, being represented by $o1 - o2 < \alpha $, $o2 - o3 < \beta $, or all the child nodes are full, then the tree expands horizontally adding the new word as being a new child node.

The output of the algorithm is a positive or a negative emotion, and the emotion can be classified into anger, disgust, fear, joy, sadness, or surprise. These emotions are measured in the studied regions, some days before and after the change in the user behavior.

At the end, the event is identified according to its topic and subtopic by the NLP and the respective emotions of the messages performed by the affective analysis. This information is useful to understand the nature of the event.

### F. Evaluation Methodology of Topic/Subtopic Detection and Affective Analysis

In this work, the topics to be analyzed by NLP were family, love, religion, study, work, friendship, politics, nature, entertainment, and health. The topic detection by NLP was compared with two other methods, the SFPM [67], and the BNgram [68] using two different and known datasets [87], the USA presidential election tweets and the Manhattan tweets. The first database contains the tweet ids with approximately 280 million tweets related to the 2016 United States presidential election. They were collected between July 13, 2016 and November 10, 2016 from the Twitter API. The other database, Manhattan, contains tweets tagged with GPS coordinates within the boundaries of the area of Manhattan in New York City, and comprises 671,170 tweets extracted during the month of December 2014.

It is important to note that all the methods, including normalization, NLP, and affective analysis, can be easily portable to any other language. Thus, the English language version was used for comparison of both datasets, the US Elections and Manhattan.

As previously stated, the emotion detection performed by the affective analysis in this work was compared with EmoMix [78] and another affective metric proposed in [70]. Both metrics generate the emotions anger, disgust, fear, joy, sadness, or surprise, and for this reason, they were chosen for the performance comparison.

### G. Evaluation of the Early Event Detection System

Two solutions were chosen for evaluation of the proposed early event detection system: the MED [39] and another event detector proposed in [40]. The same database of messages extracted during the period of emergence of COVID-19 was used for the other two event detection systems. However, it is important to note that the other methods do not extract and analyze the social user behavior. Thus, the detection system proposed in this paper cannot be compared with other similar systems because of the lack of related works that detect events through the user behavior.

### H. Tools and Processing Environment

**FIGURE 11.**
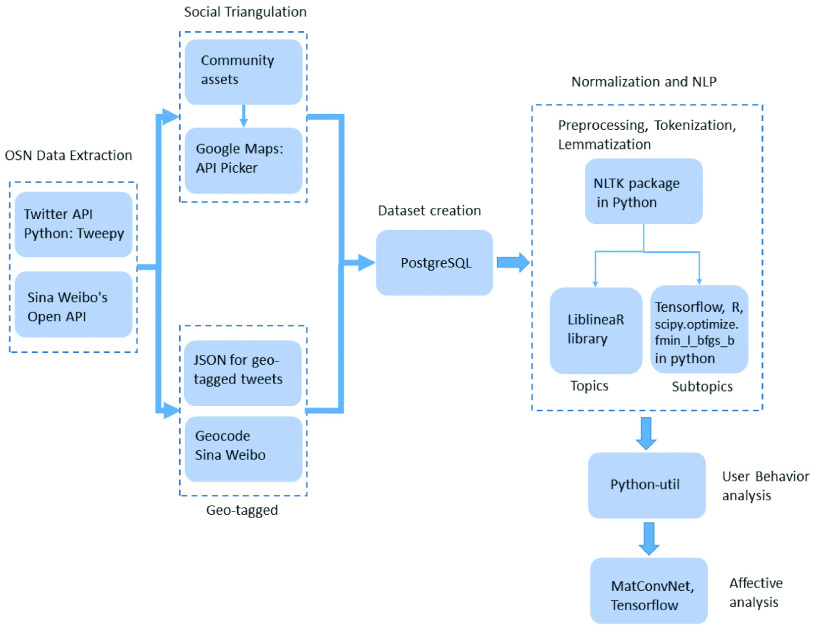
Block diagram of the main Software packages and programming languages used in each step of the proposed event detection system.

Fig. 11 shows a block diagram of all steps implemented in this work, which entirely represents the proposed event detection system. In this diagram, the information regarding the Software packages, programming languages, and processing environment is described.

As can be observed in Fig. 11, firstly, the OSN raw data extraction from Twitter and Weibo is performed. To this end, the Twitter API 2.0 and a Python library, the Tweepy 3.9.0, are used for accessing the Twitter API; similarly, the Sina Weibo’s Open API 1.4 is used for data extraction. Some data extracted from OSN are geo-tagged, these data are then treated by JavaScript Object Notation (JSON) to format user information from Twitter, and the Weibo data are treated by Geocode Sina Weibo. Thus, the data extracted from OSN are tabulated. On the other hand, the data that are not geo-tagged are analyzed by a ST technique based on the community assets of the OSN users to discover their location. To accomplish this, Google Maps with the API Picker 2.0 are used. Later, the Dataset is created and organized using the PostgreSQL Database Server 12.3.

The Normalization of data and the NLP are performed with the NLTK package in Python, in which the LiblineaR library 2.30 [85] is used to classify the messages into different topics, and the Tensorflow 2.0, R package and scipy.optimize.fmin_l_bfgs_b package 1.5.1 in Python are used for the subtopic classification, simulating the DBN model. The scipy.optimize.fmin_l_bfgs_b is used as the L-BFGS algorithm to optimize the cost function in the DBN to perform the text processing in NLP. The study of the user behavior changes is performed by the python-util package 2.0 and the affective analysis is performed by the MatConvNet [88] toolbox of MATLAB R2019a for the data trainning phase and the Tensorflow 2.0 with Python 3.5, Numpy 1.19 and Pandas 1.05 for the testing phase.

The data extracted from OSN are composed by different fields, consisting of the message id, text, username, date, hashtags, geo-location, mentions, favorites, and communities. We looked for messages, in the language of the studied regions, a variable named iso_language_code. The messages had their location confirmed by the geo-location of the messages, in which a variable called geoset was used. Thus, the messages were separated by regions, by a variable called towns. The created_at variable was used to identify the analyzed period. All the data were stored in the PostgreSQL database to organize the collected information. Later, other fields were added to the database, such as topics, subtopics, and emotions.

### I. Performance Evaluation Metrics

The performance parameters used in this work are accuracy, sensitivity or recall, F-measure, and G-mean, to assess the effectiveness of the NLP and affective analysis approach.

\begin{align*} \text {Accuracy}=&\frac {\text {TP} + \text {TN}}{\text {TP} + \text {TN} + \text {FP} + \text {FN}} \tag{4}\\ \text {Sensitivity}=&\frac {\text {TP}}{\text {TP} + \text {FN}} \tag{5}\\ {\text {F}\text {-measure}}=&\frac {{2 \times \left ({\text {precision} \times \text {recall} }\right)}}{\text {precision} + \text {recall}} \tag{6}\\ \text {G}{\text {-mean}}=&\sqrt {\text {TP}_{\text {rate}} \times \text {TN}_{\text {rate}} } \tag{7}\end{align*}

where TP, TN, FP, and FN represent true positives, true negatives, false positives, and false negatives, respectively. The $\text {TP}_{\text {rate}} = \text {TP}/p$ and $\text {TN}_{\text {rate}} = \text {TN}/n$, in which $p$ represents the number of positive samples and $n$ represents the number of negative samples. The metric called geometric mean or G-mean is a single score that helps to understand the sensitivity and specificity of data.

## IV. Results and Discussion

This section presents the main results of the ST technique to calculate the geographical location of the user, the topic and subtopic identification by the NLP, the analysis of topic changes, the affective analysis, and finally, the global performance of the proposed early detection system.

### A. Social Triangulation

Of the 8,023 users analyzed in total, only 18% configured the location in his/her profile. The other 82% had their location discovered by the ST technique.

**FIGURE 12.**
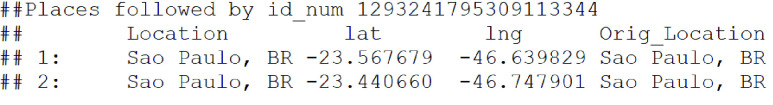
Location of the communities followed by a person according to the script used in this work.

Fig. 12 presents the geographical location of the communities followed by a person according to the output of the script used in this work. In this example, the user follows communities located next to her/his home location.

Dividing the messages and respective users according to the organization and the users they follow revealed that the majority of all users follow only one local organization. Thus, this organization was considered the unique recipient of local information.

According to the analysis of the user followers it was possible to discover the user location in the cases that the user location was not directly available in the OSN. Thus, only the messages of this user were considered. In the ST analysis, the citizen’s associations, public institutions, businesses, and media organizations were detected in their OSN.

**FIGURE 13.**
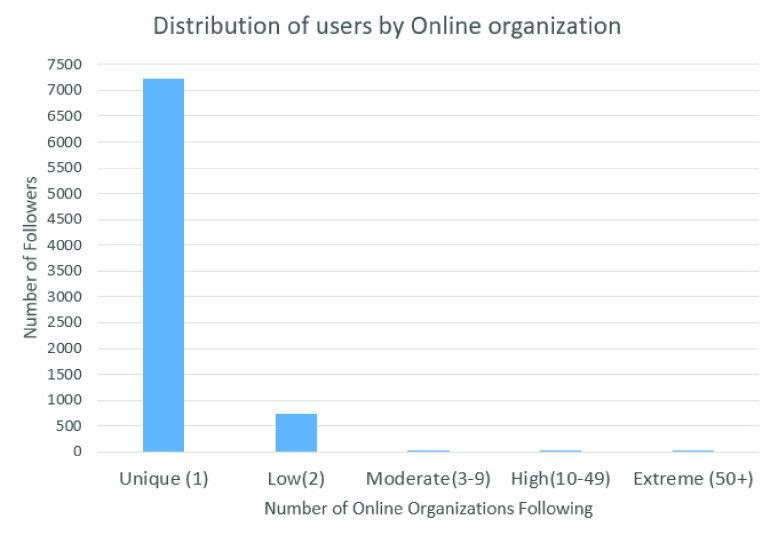
Distribution of users in relation to the number of online organizations they follow.

Fig. 13 shows the distribution of the previously defined five types of users that are grouped according to the number of online local organizations that the users follow.

It can be seen in Fig. 13 that 90% of the users follow only one local organization, named “Unique” in the graph. 9.2% of the users follow only two organizations, named “Low” in the graph. 0.3% follow from three to nine organizations, named “Moderate”. 0.1% of the users follow 10 to 49 organizations, named “High”, and finally, 0.3% of the users follow more than 50 organizations in OSNs, named “Extreme.”

This distribution indicates that a minority of users, grouped in the moderate, high, and extreme types, receive multiple information from diverse communities, in which those organizations represent the location of the user’s home, work, or other places where the user has been walking around. Conversely, the majority of users correspond to the unique or low types, which directly receive information from only one or two organizations that commonly represent the user’s home [34] or work. Hence, the information about some organizations helps to detect the user’s location, and the user has to follow at least one organization to determine the user location.

Of all the people analyzed by the ST technique, the majority are local to a state, and the others identify their location in relation to a municipality.

As already stated, in order to evaluate the ST performance, the location information of the users who had configured their locations (18% of the all users analyzed) was compared with the results obtained by the ST technique. Thus, of these 17.99% (1,444 users), a total of 1,343 users (93.005%) had their geographical location correctly determined by the performed ST technique.

### B. Topics/Subtopic Identification by NLP

In this subsection, the experimental results regarding the topic and subtopic identification are presented.

For this task, the complete dataset of the study was used. The 10-fold cross validation was used during both the training and testing phases. For the topic identification, the dataset was used and split into 80% for training and 20% for testing.

The DBN configuration used in this work consists of four layers, in which the unit numbers of each layer are 2000-1000-500-6, running for 1000 pre-training epochs, and considering a learning rate of 0.01. For the L-BFGS algorithm, a penalty factor $\lambda $ and the number of previous computations $m$, introduced in (3), were set to 2 and 6, respectively.

Table 3 shows the average results for accuracy, sensitivity, F-measure, and G-mean for the classification of all topics in the training and testing phases. As mentioned above, the topic identification consists of ten predefined classes: family, love, religion, study, work, friendship, politics, nature, entertainment, and health. Due to the high number and diversity of subtopics in all regions, these are not presented, and only the COVID-19 subtopic is analyzed.

**TABLE 3:** Accuracy, Sensitivity, F-Measure, and G-Mean Results for Topic Identification by NLP in the Training/Testing Phase

Class	Accuracy	Sensitivity	F-measure	G-mean
family	0.90/0.89	0.92/0.92	0.90/0.90	0.90/0.90
love	0.89/0.88	0.91/0.90	0.89/0.88	0.89/0.88
religion	0.92/0.91	0.91/0.90	0.91/0.90	0.91/0.90
study	0.91/0.90	0.91/0.90	0.91/0.90	0.91/0.90
work	0.89/0.88	0.90/0.89	0.89/0.88	0.89/0.88
friendship	0.89/0.87	0.90/0.87	0.89/0.87	0.88/0.87
politics	0.91/0.90	0.91/0.90	0.91/0.90	0.90/0.89
nature	0.88/0.87	0.89/0.88	0.88/0.87	0.89/0.88
entertainment	0.87/0.85	0.89/0.88	0.87/0.86	0.89/0.88
health	0.93/0.92	0.92/0.91	0.92/0.91	0.90/0.89

Regarding the subtopic classification, Table 4 shows the average values of the following performance metrics: accuracy, sensitivity, F-measure, and G-mean for the subtopic identification in the training and testing phases.

**TABLE 4:** Accuracy, Sensitivity, F-Measure, and G-Mean Results for the Subtopic Identification by NLP

Class	Accuracy	Sensitivity	F-measure	G-mean
Training	0.90	0.88	0.88	0.89
Testing	0.87	0.91	0.89	0.92

The topic/subtopic detection by NLP was compared with the other two methods, the SFPM [67], and the BNgram [68]. These methods had their parameters set according to [66], [68].

To validate the proposed method of topic selection, three datasets were used; the US Elections, US Manhattan datasets, and our dataset about COVID-19 symptoms.

Table 5 shows the comparison of the topic detection algorithms, in terms of accuracy, sensitivity, F-measure, and G-mean for the US Elections dataset, for the politics topic.

**TABLE 5:** Performance Parameter Results for the Topic Identification in the US Elections Dataset

Class	Accuracy	Sensitivity	F-measure	G-mean
Proposed	0.87	0.87	0.87	0.85
SFPM [67]	0.68	0.68	0.68	0.68
BNgram [68]	0.65	0.65	0.65	0.65

Table 6 shows the comparison of the topic detection algorithms using the same performance evaluation parameters presented in Table 5 for the Manhattan dataset. The entertainment topic and the “Bocelli Concert” subtopic were used as specific cases. It is important to note that both the SFPM [67] and BNgram [68] methods have a limitation for the subtopic classification task.

**TABLE 6:** Performance Parameter Results for the Topic Identification in the US Manhattan Dataset

Class	Accuracy	Sensitivity	F-measure	G-mean
Proposed	0.86	0.85	0.85	0.85
SFPM [67]	0.71	0.69	0.69	0.69
BNgram [68]	0.69	0.68	0.68	0.68

Table 7 shows the comparison of the topic detection algorithms, in terms of accuracy, sensitivity, F-measure, and G-mean for our dataset, for the health topic.

**TABLE 7:** Performance Parameter Results for the Topic Identification in the COVID-19 Symptoms Dataset

Class	Accuracy	Sensitivity	F-measure	G-mean
Proposed	0.93	0.92	0.92	0.92
SFPM [67]	0.54	0.52	0.52	0.52
BNgram [68]	0.52	0.51	0.51	0.51

It can be observed from Tables 5, 6 and 7, the proposed NLP technique achieved the best performance parameter values in relation to the SFPM [67] and the BNgram [68]. This means that the subtopic identification by the proposed method is highly reliable, and this will help to better describe this kind of event.

### C. Time Period to Change the Topics and the User Behavior Change

In the experimental tests, at a specific moment, the frequency of posted messages changed for different topics. Therefore, the topics and subtopics were determined in advance.

**FIGURE 14.**
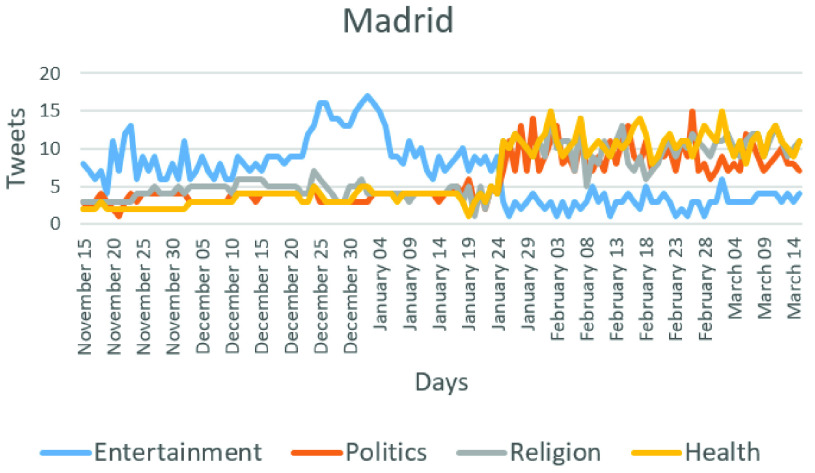
Number of Tweet messages and main topic changes across days in the city of Madrid.

Each city has different characteristics in terms of impacted period and prevalent topics that are spread as events. For example, the frequency of posting increased, on average, from 15 to 30 messages per day close to the time when the event was spread in the OSN in the region of Madrid as depicted in Fig. 14. The volume of the messages increased rapidly following the arrival of an event. The topics began to change from entertainment to religious, politics, and health. Thus, Fig. 14 shows a change in the users’ behavior after January 24th. The other six topics are not shown because they present a stability in the number of tweets during the period of study. It is important to note that on January 31st, the first case with COVID-19 symptoms was confirmed in Spain.

If one or more topics have their number of message changed, in other words, there is a decrease or increase during some hours or a day [89], [90], it indicates a change in the user behavior in OSN. In that case, a new event is considered. Based on the analysis of our collected data, an increase or decrease of 30% in the number of messages for a topic is considered a threshold that indicates an event detection.

**FIGURE 15.**
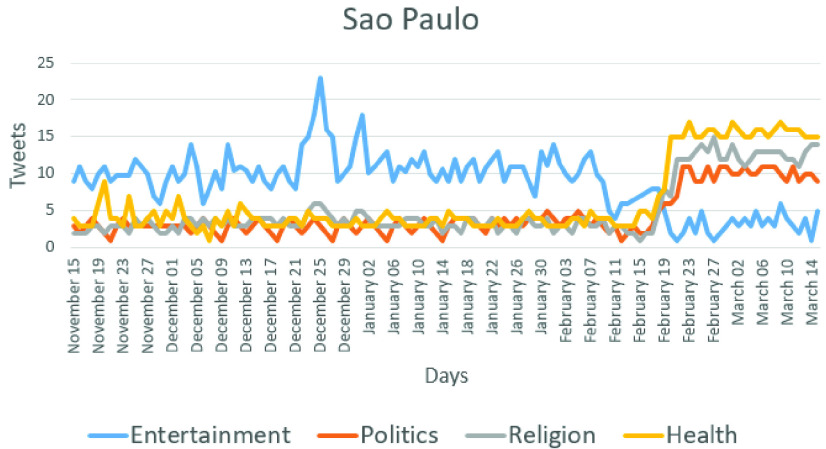
Number of Tweet messages and main topic changes across days in the city of Sao Paulo.

The characteristics of the Twitter users in the city of Sao Paulo in Brazil are presented in Fig. 15. This city was chosen because on average, more messages were posted there per day than in the other cities. As can be observed, on average, the frequency of messages increased from 19 to 42 messages per day close to the event. Fig. 15 shows a change in the users’ behavior after February 20th, and the first confirmed case was recorded in February 26th. In Fig. 14 and Fig. 15, the health topic increases by the pandemic, but the politics topic also increases because it is associated with public health.

Fig. 14 and Fig. 15 also show the exact date when the users’ behavior changed in different regions. It is worth noting that changes in the users’ behavior can be useful in order to detect some events, and more importantly, the nature of that event can be unknown and automatically detected.

The user behavior in other cities and countries had a similar trend related to the COVID-19 subtopic. The topics and subtopics of a few user posts began to change, and then, the behavior of other users located in the same region started to change, similar to the arrival of the new event.

**FIGURE 16.**
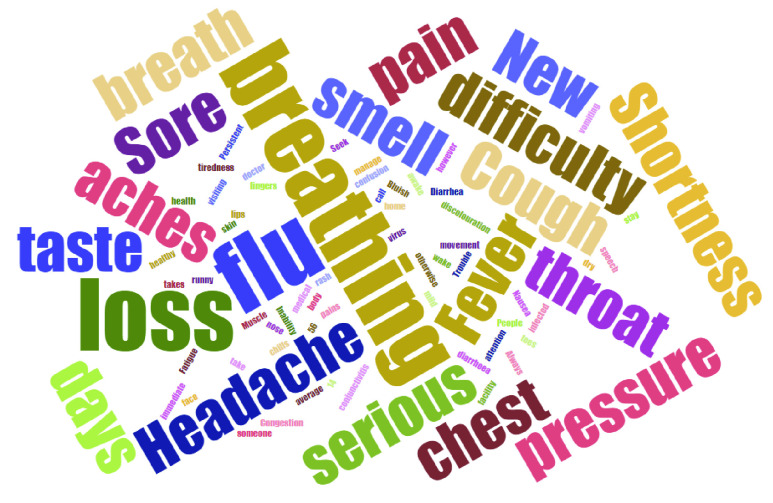
Word cloud collected near the event of COVID-19 in New York City.

Fig. 16 shows a word cloud that contains the keywords found in the tweets, in New York City, USA. The other cities have similar keywords. It can be noted that the event has a relation to flu, respiratory crisis, and other health problems related to the COVID-19 disease symptoms.

### D. Affective Analysis Using Tree-CNN

In the experiments, the following Tree-CNN configuration was used: the maximum depth of the tree is 3, the threshold defined by the tree size $\alpha = 0.1$, and the other threshold defined by the user $\beta = 0.1$; and the maximum number of child nodes for a branch node is set at 5, 10, 20. The networks are trained using mini-batch stochastic gradient descent, a fixed momentum of 0.9, and a weight decay with $\gamma = 0.001$. The CNNs were trained using 50, 100, 200, and 300 epochs, of which 300 epochs got the highest accuracy. The learning rate was 0.1.

Table 8 shows the average results of accuracy, sensitivity, prediction, F-measure, and G-mean for the detected emotions in the training phase of the Tree-CNN algorithm.

**TABLE 8:** Accuracy, Sensitivity, F-Measure, and G-Mean Results for the Detected Emotions in the Training Phase

Class	Accuracy	Sensitivity	F-measure	G-mean
Anger	0.91	0.91	0.91	0.90
Disgust	0.89	0.90	0.89	0.89
Fear	0.91	0.91	0.91	0.90
Enjoy	0.89	0.89	0.89	0.89
Sadness	0.91	0.91	0.91	0.91
Surprise	0.89	0.88	0.88	0.89

Table 9 shows the average results of accuracy, sensitivity, F-measure, and G-mean for the detection emotion task using the Tree-CNN algorithm in the testing phase.

**TABLE 9:** Accuracy, Sensitivity, F-Measure, and G-Mean Results for the Detected Emotions in the Testing Phase

Class	Accuracy	Sensitivity	F-measure	G-mean
Anger	0.90	0.90	0.90	0.89
Disgust	0.88	0.90	0.88	0.88
Fear	0.90	0.91	0.90	0.90
Enjoy	0.88	0.91	0.89	0.89
Sadness	0.89	0.90	0.89	0.89
Surprise	0.87	0.86	0.86	0.85

Table 10 shows the average results for accuracy and F-measure for the detected emotions in the testing phase for the proposed method and for other machine learning algorithms, such as the SVM, RF, DT, and CNN.

**TABLE 10:** Accuracy and F-Measure (Accuracy/F-Measure) Results for the Detected Emotions in the Testing Phase

Method	Anger	Disgust	Fear	Enjoy	Sadness	Surprise
Tree-CNN	0.90/0.90	0.91/0.92	0.90/0.92	0.93/0.94	0.90/0.93	0.90/0.93
SVM	0.80/0.81	0.79/0.81	0.81/0.81	0.83/0.82	0.84/0.83	0.84/0.83
RF	0.82/ 0.82	0.80/0.82	0.82/0.81	0.83/0.84	0.85/0.85	0.85/0.85
DT	0.83/0.82	0.81/0.82	0.83/0.82	0.82/0.83	0.84/0.83	0.84/0.83
CNN	0.85/0.84	0.82/0.84	0.84/0.85	0.84/0.84	0.86/0.86	0.86/0.86

As can be observed from Table 10, the Tree-CNN algorithm reached the highest F-measure scores for each emotion compared with the other machine learning algorithms used in the current literature.

Additionally, the proposed affective metric was compared with other two metrics, the EmoMix [78] and another method proposed in [70]. Table 11 presents the average results of accuracy and the F-measure for these affective metrics.

**TABLE 11:** Accuracy and F-Measure (Accuracy/F-Measure) Results for the Proposed Affective Metric and the Related Affective Metrics

Method	Anger	Disgust	Fear	Enjoy	Sadness	Surprise
Proposed	0.90/0.90	0.91/0.92	0.90/0.92	0.93/0.94	0.90/0.93	0.90/0.93
EmoMix	0.79/0.78	0.74/0.76	0.74/0.80	0.77/0.79	0.73/0.79	0.78/0.77
Metric [70]	0.74/0.72	0.73/0.74	0.71/0.73	0.74/0.76	0.75/0.74	0.74/0.76

It is important to note that the negative messages represent events with a potential problem, and this fact requires more attention from the corresponding authorities but also from the wider audience.

**FIGURE 17.**
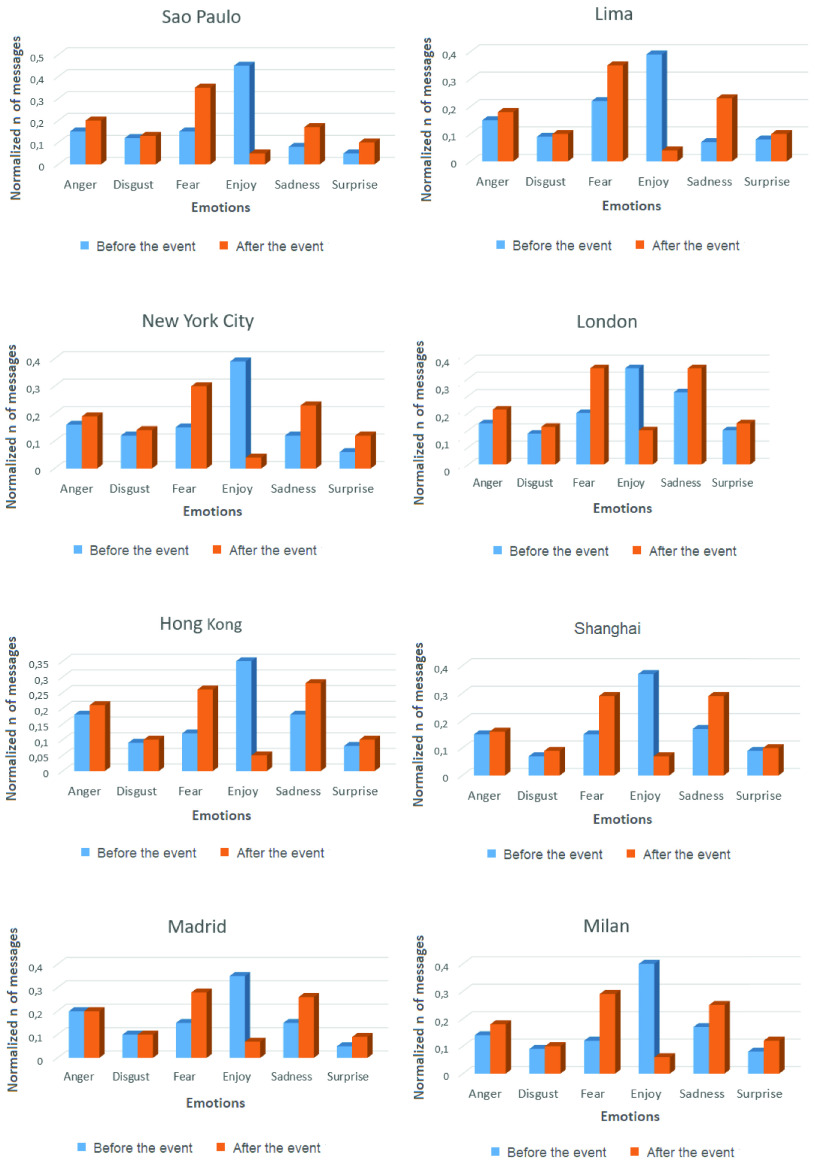
Normalized number of messages for each classified emotion in the cities of Sao Paulo, Lima, New York City, London, Hong Kong, Shanghai, Madrid, and Milan during 15 days before and 15 days after the event of COVID-19.

Fig. 17 shows the emotion types presented in all the analyzed regions, 15 days before and 15 days after the appearance of the COVID-19 event in each region. The numbers of emotions presented in the OSNs depicted in the graphs are normalized for a better visualization. As can be observed from Fig. 17, the number of messages corresponding to negative emotions, such as fear and sadness, have a significant increase in the later date analyzed.

### E. Evaluation of Discovered Events

This work evaluated the opportunity and effectiveness to detect events using OSN data. Thus, the proposed detector was compared with other two methods, the MED [39] and the another event detector reported in [40].

**FIGURE 18.**
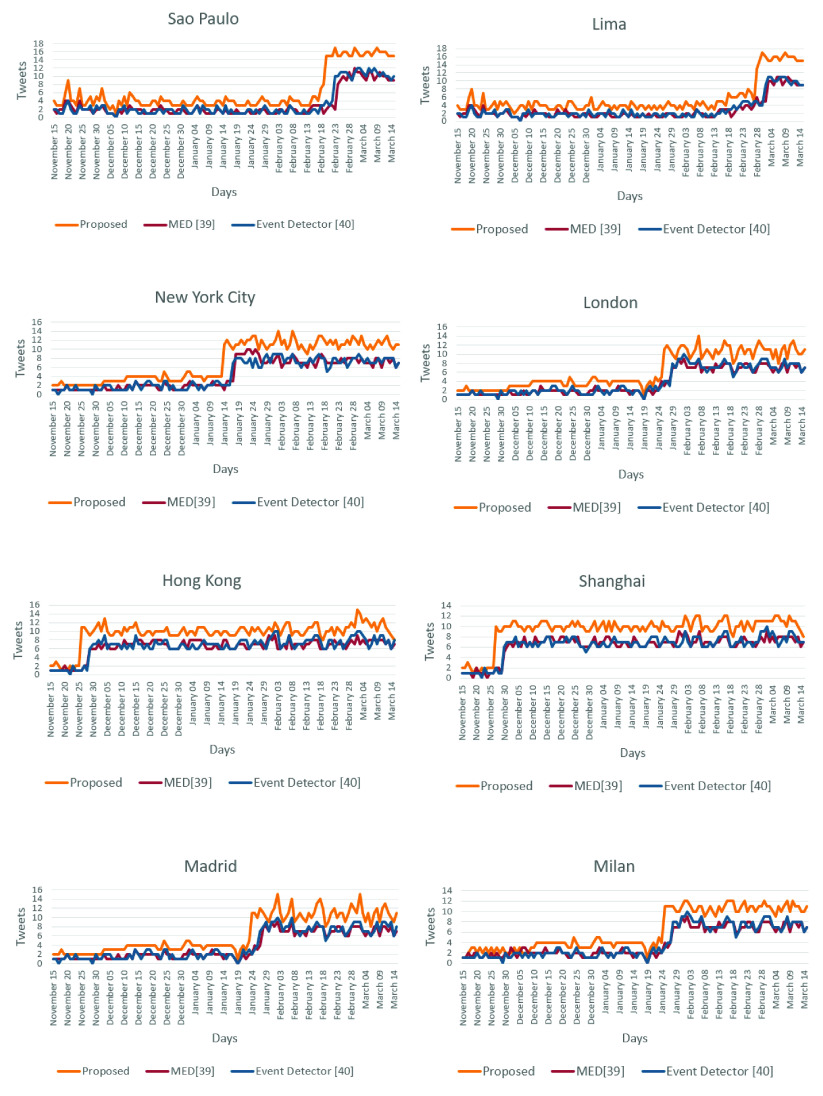
Number of Tweets by day for each method in the cities of Sao Paulo, Lima, New York City, London, Hong Kong, Shanghai, Madrid, and Milan for the health topic.

Fig. 18 presents the number of tweets by day for each method in the cities of Sao Paulo, Lima, New York City, London, Hong Kong, Shanghai, Madrid, and Milan for the health topic. The MED [39] and the Event Detector [40] methods detect an event through a large number of repeated terms presented in the messages, which consume more time for data analysis, inserting a delay for the detection of an event.

Table 12 shows the comparison of event detection algorithms and the proposed solution in terms of accuracy, sensitivity, F-measure, and G-mean using the dataset built for the study and only considering the specific subtopic related to COVID-19.

**TABLE 12:** Performance Parameter Results for the COVID-19 Subtopic, Using the Dataset Built for the Study

Class	Accuracy	Sensitivity	F-measure	G-mean
Proposed	0.92	0.93	0.92	0.93
MED [39]	0.71	0.73	0.71	0.71
Event Detector [40]	0.63	0.65	0.63	0.63

It can be observed from Table 12 that the proposed solution achieved the best performance assessment parameter values in relation to the methods introduced in [39] and [40]. Hence, the subtopic identification by the proposed method is accurate and well describes the event. It is noteworthy that the proposed system identifies the topic and each specific subtopic, being thus a more complete early event detection system. In addition, it is important to highlight that the proposed solution, according to the experimental results obtained by using the dataset built for this study, is able to detect an event, on average,

three days earlier than the other two evaluated methods [39], [40] used for performance comparison. For example, experimental results demonstrated that our proposed system was able to detect the event of interest in the city of Sao Paulo on February 20th as shown in Fig. 15, whereas the systems proposed in [39] and [40] detected the same event for the same city on February 24th and February 23rd, respectively. Similar results were obtained in the rest of the cities under study. Table 13 presents the number of days that our proposed system and the systems used for performance comparison were able to detect before the first event was officially confirmed in each studied city.

**TABLE 13:** Number of Days That Our Proposed System and Those Proposed in MED [39] and [40] Were Able to Detect Before of the First Confirmed Event in Each Studied City

Region	Proposed	MED [39]	Event Detector [40]
Lima	7	3	4
New York	6	2	3
Sao Paulo	6	2	3
Hong Kong	5	2	2
Shanghai	4	1	1
London	5	2	2
Madrid	7	3	4
Milan	6	3	3

As can be observed in Table 12 and Table 13, our proposed event detection system reached the best performance in accuracy and response time, which are very relevant performance parameters in this type of solutions.

## V. Conclusion

This work introduced and validated an event detection system at an early stage based on the user behavior information extracted from OSNs, highlighting the relevance of incorporating the user behavior change analyses into solutions of this kind.

This proposed system is agnostic about the topic of a possible event; however, our case study focused on the COVID-19 pandemic event to stress the usefulness of this solution type. Although a case on a health topic was used, the proposed system can be extended to other areas, not limiting its use to a specific topic or case. In general, the experimental results obtained demonstrate that users clearly react when some events occur. This reaction is reflected in the number of posted messages and the message topics. Therefore, tracking the user behavior in OSNs permits to identify events in specific regions, and at the beginning of the event. This work considered eight big cities around the world and a considerable amount of diverse data from different cultures. The proposed event detection system was composed of different modules, and each module of the solution was evaluated and found to have an accuracy higher than the related works referred to in the study. Thus, the technique to discover the user location, the NLP algorithm for topic and subtopic identification, and the affective analysis to discover the emotions of the messages were validated.

The case of the COVID-19 pandemic was studied, and keywords like flu, fever, and respiratory crisis, among others, were identified in the subtopic called respiratory disease. Thus, this work showed the importance of the subtopic identification by the NLP algorithm using an unsupervised machine learning technique and the use of affective analysis. According to the global results presented in Table 12 and 13, the proposed system presents a better performance than two similar event detector solutions proposed in [39] and [40]. Although cities from different countries were analyzed, a similar behavior was detected by the change in topics, but at different dates. In our case study, the COVID-19 pandemic, the message topics about health, religion, and politics emerged with more notoriety, and conversely, the number of messages regarding the entertainment topic decreased. As a topic of future work, the objective is to explore the usefulness of user behavior information in OSNs in order to detect events belonging to different topics, and a further aim is to test another deep learning algorithms to improve the system performance.
